# Endocrine disruptors, insulin resistance, and diabetes

**DOI:** 10.55730/1300-0144.6119

**Published:** 2025-10-26

**Authors:** Meriç COŞKUN, Ethem Turgay CERİT, Tuğba BARLAS, Alev EROĞLU ALTINOVA

**Affiliations:** Division of Endocrinology and Metabolism, Department of Internal Medicine, Faculty of Medicine, Gazi University, Ankara, Turkiye

**Keywords:** Endocrine disruptors, insulin resistance, diabetes, T2D

## Abstract

Endocrine-disrupting chemicals (EDCs) are compounds that interfere with hormone synthesis, secretion, metabolism, or excretion. Evidence indicates that increased exposure to EDCs is associated with insulin resistance and, most notably, type 2 diabetes worldwide. This suggests a diabetogenic effect that is independent of obesity, underscoring the complex mechanisms and broad impact of EDCs on metabolic health. Key pathways include hormone mimicry and antagonism, altered hormone metabolism, inflammatory responses, and mitochondrial dysfunction. This review summarises the mechanisms through which EDCs contribute to these conditions and evaluates the epidemiological and experimental evidence supporting these associations.

## 1. Introduction

Endocrine-disrupting chemicals (EDCs) are substances that interfere with hormone synthesis, secretion, metabolism, or excretion. They include synthetic compounds, industrial solvents, lubricants, dioxins, plasticisers, phthalates, pesticides, and pharmaceuticals [1]. EDCs are designed to have long half-lives, making them resistant to breakdown or metabolism. Consequently, they can persist for many years in soil, water, and animals and plants within the food chain, as well as in human tissues and secretions [2]. The uncertainty in the latency between exposure and clinical effects, the likelihood of simultaneous exposure to multiple EDCs, the absence of well-defined dose–response relationships, and potential epigenetic influences all contribute to the challenges of predicting their harmful effects.

The diabetogenic hypothesis suggests that EDCs are potential risk factors for metabolic syndrome and type 2 diabetes (T2D), independent of obesity [1,3]. The associations between EDCs, insulin resistance (IR), T2D, and adverse metabolic outcomes appear to be dose-dependent. Moreover, although less common than in T2D, EDCs may also contribute to type 1 diabetes (T1D) by inducing beta-cell damage [4]. This review summarises the mechanisms through which EDCs promote these conditions and evaluates the epidemiological and experimental evidence supporting these associations.

## 2. Mechanisms by which EDCs contribute to IR and T2D development

### 2.1. Hormone mimicry and antagonism

EDCs can imitate natural hormones by binding to hormone receptors and either activating or blocking their normal functions. Bisphenol A (BPA), which competes with 17-beta oestradiol, can bind to oestrogen receptors (ERs), disrupting normal hormone signalling and glucose metabolism. This mimicry can cause altered gene expression and metabolic dysfunction. Activation of ERs in the liver impairs insulin signalling and increases gluconeogenesis, leading to hyperglycaemia [5].

### 2.2. Alteration of hormone metabolism

EDCs like phthalates can interfere with hormone metabolism, affecting synthesis, breakdown, and transport. Phthalates activate peroxisome proliferator-activated receptors (PPARs), particularly PPARγ, involved in adipogenesis and lipid metabolism. Activation of PPARγ by phthalates results in increased fat storage and altered glucose metabolism, thus promoting IR [6].

### 2.3. Change of glucose transporter (GLUT) expression

Dioxin or 2,3,7,8-tetrachlorodibenzo-p-dioxin (TCDD), a persistent organic pollutant (POP), affects glucose transport activity in adipose tissue, the pancreas, and the brain. This results in hypertriglyceridemia, early atherosclerosis and loss of beta cell function [7].

### 2.4. Disruption of receptor expression

EDCs can also affect the expression and sensitivity of hormone receptors. Polychlorinated biphenyls (PCBs) are known to decrease insulin receptor expression on cell surfaces, impairing the body’s ability to respond to insulin and leading to IR [8].

### 2.5. Insulin-like growth factor (IGF) system

The IGF system, crucial for growth and metabolic regulation, is another target for EDCs. Disruptions in IGF signalling due to EDC exposure can lead to metabolic disorders, including IR and diabetes. EDCs like dioxins, phthalates, and BPA have been shown to disrupt IGF signalling. [9].

### 2.6. Inflammatory pathways

EDCs may cause IR by increasing chronic low-grade inflammation. PCBs increase tumour necrosis factor-alpha (TNF-α) and interleukin-6 (IL-6), BPA decreases serum adiponectin levels and increases serum IL-6 and TNF-α, leading to IR and glucose intolerance [10].

### 2.7. Increase in reactive oxygen species (ROS)

BPA leads to T2D in insulinoma cell lines (INS-1 cells), resulting in elevated ROS levels, increased apoptosis, and reduced cell viability [11]. PCBs, which are POPs, impair glucose metabolism by activating ROS/NF-κB signalling and downregulating HNF1b [12]. In Langerhans cells obtained from rats, exposure to tributyltin (TBT) led to increased apoptosis, decreased cellular viability, and elevated ROS levels [13]. Diethylhexyl phthalate (DEHP) metabolites increased malondialdehyde (MDA) levels, an oxidative stress biomarker, in older adults. This increase in MDA led to IR via the oxidative stress pathway [14].

### 2.8. Inflammation of the islet of Langerhans

BPA exposure increases T1D in both adult nonobese diabetic (NOD) female mice and female pups through transmaternal exposure [15].

### 2.9. Nonalcoholic fatty liver disease (NAFLD)

EDCs such as dioxins and BPA may trigger NAFLD through hepatotoxic effects or by inducing systemic or hepatic IR [16].

### 2.10. Mitochondrial dysfunction

Mitochondrial dysfunction, apoptosis, and excessive production of reactive oxygen species are mechanisms that contribute to the development of T2D [17]. Experimental studies indicate that chemicals like BPA, benzyl butyl phthalate (BBP), TCDD, diethylstilbestrol (DES), TBT, perfluorooctanoic acid (PFOA), perfluorooctane sulfonic acid (PFOS), atrazine, and cadmium may induce the development of T2D through mitochondrial dysfunction via these pathways [17].

### 2.11. Combined impact of EDCs

The compounding effects of multiple EDCs, including BPA, phthalates, and PCBs, lead to greater disruption of glucose metabolism and higher IRs than individual EDC exposure. In children, exposure to EDCs like pesticides, PCBs, and BPA has been linked to IR and related metabolic disorders. Although data on children are limited, existing evidence suggests significant health risks from EDC exposure. Studies indicate a statistical link between EDC exposure and IR in children, underscoring the need for more research in this area [18].

The figure (Figure) summarises the notable mechanisms of adverse impacts of the EDCs on the occurrence of diabetes.

## 3. Evidence from animal and human epidemiological studies

### 3.1. Bisphenol A (BPA) (C15H16O2)

Animal studies show that BPA exposure induces oxidative stress and β-cell apoptosis, and impairs glucose-stimulated insulin release, leading to glycolipotoxic damage [11,19]. In NOD mice, BPA exposure results in insulitis and autoimmune features of T1D [5,20]. Human epidemiological studies support these findings, with several meta-analyses linking urinary and serum BPA levels to IR and T2D [21], and elevated BPA concentrations have also been reported in children with T1D [22]. Associations with gestational diabetes mellitus (GDM) are inconsistent. While some studies suggest BPA may contribute to GDM [23], others report no correlation with impaired glucose tolerance during pregnancy [24]. Overall, the evidence is strong for IR and T2D, moderate for T1D and GDM, but causal inference is limited by cross-sectional study design.

### 3.2. Tetrabromobisphenol A (TBBPA)

Experimental studies demonstrate that TBBPA impairs mitochondrial function and induces apoptosis in pancreatic β-cells [25]. Human studies are scarce and inconsistent; one report suggested that combined BPA/TBBPA exposure was inversely associated with GDM risk, whereas bisphenol S (BPS) was positively associated [26]. Mechanistic plausibility is strong, but epidemiological evidence remains insufficient.

### 3.3. Phthalates

Phthalates are liquid plasticisers. Exposure to phthalates comes from food, personal care products and air [27]. Rodent studies reveal that phthalates exacerbate diabetes by activating PPARγ, impairing GLUT-2, and disrupting insulin signalling, often with sex-dependent differences [28,29]. For example, di-(2-ethylhexyl) phthalate worsened hepatic insulin sensitivity in female T2D mice, while dibutyl phthalate impaired insulin secretion and glucose tolerance by downregulating PI3K/AKT signalling and GLUT-2 [28]. Human studies, including the National Health and Nutrition Examination Survey (NHANES), demonstrate dose–response associations between urinary phthalates and T2D [27,30], although inconsistencies remain due to biomarker variability and confounding factors. While exposure to phthalates has been shown to decrease pancreatic insulin content and cause abnormal remodelling of beta cells in animal studies [31], epidemiological studies suggest that establishing a causal link between T1D and phthalate exposure is difficult [32]. Overall, the evidence is moderate to strong, but mechanistic confirmation in humans is still needed.

### 3.4. Persistent organic pollutants (POPs)

POPs are chemicals that resist degradation and can accumulate in adipose tissue [33]. Exposure to POPs in adult mice impairs the ability of insulin to stimulate Akt phosphorylation and muscle glucose uptake in a dose-dependent manner. Conversely, at low doses, visceral fat accumulation, TNFα mRNA levels in adipose tissue, and macrophage infiltration were decreased [34]. Although inconsistent results have been observed, POPs, especially mixtures of organochlorine pesticides (OCPs) and PCBs, increase the risk of IR and T2D [35].

PCBs are divided into dioxin-like PCBs (DL–PCBs) and nondioxin-like PCBs (NDL–PCBs) [36]. In a historical cohort, exposure to DL–PCBs was associated with a high incidence of T2D [37]. The high heterogeneity in the results makes it challenging to make judgments about all PCBs based solely on epidemiological studies.

### 3.5. Dioxin

TCDD is primarily produced as industrial waste but can also be generated during natural disasters. Dioxin may be found at high levels in soil, sediments and foods, especially dairy products, meat, fish and shellfish [38]. In animal experiments, short-term exposure to TCDD improves insulin sensitivity [39], whereas chronic exposure induces IR and diabetes [40], highlighting the complexity of the dose–response relationship. Human meta-analyses generally support a positive association between serum dioxin levels and T2D [41], although heterogeneity across populations persists. Together, these findings suggest relatively strong evidence but with significant differences between acute and chronic exposure effects.

### 3.6. Perfluoroalkyl acids (PFAS)

PFAS, such as PFOS and PFOA, are used as synthetic chemicals for thermal stability [42]. PFAS exposure in NOD mice leads to a reduction in phospholipids, an increase in lithocholic acid, and enhanced insulitis [43]. Exposure to PFAS and PFOS increases fasting insulin, fasting glucose or Homeostatic Model Assessment of Insulin Resistance (HOMA-IR) [44]. An association was found between serum PFAS levels and the risk of prediabetes [45]. There was no clear evidence that PFAS cause diabetes, but PFAS exposure increased impaired glucose homeostasis and GDM, particularly in normal-weight pregnant women [46]. Animal studies consistently show that PFAS disrupt mitochondrial function and increase insulitis in NOD mice. In humans, PFAS exposure has been linked to impaired glucose tolerance and GDM, though associations with T2D remain inconsistent. Differences in PFAS subtype, half-life, and study design may explain this variability [42–46].

### 3.7. Tributyltin (TBT)

Tributyltin ((C 4 H 9) 3 Sn +) is used as an antifungal, wood preservative, and colourant in marine vessels. Therefore, it is the most important pesticide found in marine and freshwater systems. Due to its high fat solubility, it bioaccumulates in organisms higher up the food chain, and TBT is detected in food, blood, and tissues. Studies observed that the pancreatic beta- and alpha-cell mass decreased in male mice injected with TBT, leading to increased hepatic gluconeogenesis, which, in turn, caused glucose intolerance and IR [47]. Oral TBT administration in rats increases fasting serum insulin and adiponectin levels and decreases glucagon levels by affecting the insulin receptor signalling cascade and GLUT-4 expression in both skeletal muscle and liver. While organotins have been shown to cause diabetes by reducing insulin secretion in animal studies, there are insufficient epidemiological studies on this topic [48].

### 3.8. Dichlorodiphenyltrichloroethane (DDT)

DDT is a common environmental pollutant widely used in both agriculture and malaria control, with well-documented effects on oestrogen receptor (ER) function. DDT increases the risk of diabetes, especially in Asian patients [49]. In animal studies, the mechanism is not clear, although results suggest that DDT may cause diabetes through mitochondrial dysfunction or insulin secretion defects [50]. It was also observed that chronic exposure to the DDT metabolite dichlorodiphenyltrichloroethane (DDE) in NOD mice increased the incidence of T1D by decreasing regulatory T cells and suppressing IL-4 and IL-10 [51]. However, these animal study results have not been supported by epidemiological studies [32]. The persistent nature of these chemicals complicates causal inference, as exposure is historical and widespread.

### 3.9. Diethylstilbestrol (DES)

DES is an active oral oestrogen. In mice, prenatal DES exposure was not associated with the development of diabetes, although it increased insulin [52].

### 3.10. Heavy metals

Arsenic (As) occurs naturally in soil, rocks, and sediments and can also be released during agricultural and mining activities. Cigarette smoke contains As, and smoking can double the daily intake of this element [53]. Arsenic reduces adipogenesis and increases lipolysis in white adipose tissue. Exposure to As through digestion can lead to the development of diabetes via the sirtuin 3 (SIRT3)-forkhead box O3 (FOXO3a) pathway [54].

Cadmium (Cd) is a heavy metal found in high amounts in mines and industrial wastes [55]. Chronic Cd exposure can result in hyperglycaemia, decreased insulin sensitivity in muscle and liver, impaired lipid metabolism, and reduced leptin levels [56]. Cd exposure dose-dependently increases the risk of prediabetes and diabetes [57].

Both arsenic and cadmium have been shown in animal models to induce oxidative stress and mitochondrial dysfunction. Human studies strongly support an association between chronic arsenic exposure and T2D, while evidence for cadmium is inconsistent and less robust. Thus, the evidence base is strong for arsenic but weaker for cadmium, although both share common mechanistic pathways [53–57].

## 4. Future directions

There are several challenges in assessing the effects of EDCs on glucose homeostasis, IR, and the development of diabetes. Key factors include the wide structural diversity of compounds to which humans are exposed, the need to measure chemicals in metabolically relevant tissues, the presence of multiple mechanisms of action for a single chemical, the influence of metabolites, and potential interactions between different chemicals. In addition, individual genetic susceptibility, variations in EDC target genes, differences in genes regulating EDC metabolism, concurrent diabetes risk factors, and hormonal status and sex-related effects are important considerations [58]. Although establishing direct causal relationships between EDC exposure and metabolic health remains challenging, it is essential to monitor exposures and identify high-risk groups [59]. Advances in omics technologies, including genomics, proteomics, and metabolomics, offer promising tools for exploring the mechanisms underlying EDC-induced metabolic dysfunction [1]. These approaches may enhance our understanding of the interactions between EDCs and biological systems and support the development of preventive and personalised interventions.

## 5. Conclusion

EDCs play a substantial role in the development of IR and T2D. The strongest and most consistent evidence comes from studies on bisphenols (particularly BPA), phthalates, and PCBs, which have been shown to induce oxidative stress, β-cell apoptosis, and chronic low-grade inflammation. Both experimental and epidemiological data support these mechanisms. By contrast, evidence regarding PFAS, DDT/DDE, arsenic, and cadmium remains heterogeneous and sometimes contradictory, reflecting methodological differences in exposure assessment and population variability. Future research should focus on large, longitudinal human cohorts that integrate mechanistic insights with clinical outcomes, especially in vulnerable groups such as pregnant women and children. Multiomics approaches (genomics, metabolomics, epigenomics) will be crucial for identifying biomarkers of susceptibility and clarifying gene–environment interactions. In addition, more work is required to evaluate the cumulative and synergistic effects of multiple EDC exposures, as real-life exposure is rarely to a single compound. From a public health perspective, preventive measures and regulatory policies are urgently needed. These include restrictions on BPA and phthalates in consumer products, systematic monitoring of persistent pollutants such as PCBs and dioxins, and education campaigns targeting high-risk populations. Coordinated international guidelines and effective surveillance programs will be essential to reduce the long-term metabolic burden of EDCs and to safeguard future generations.

## Figures and Tables

**Figure:**
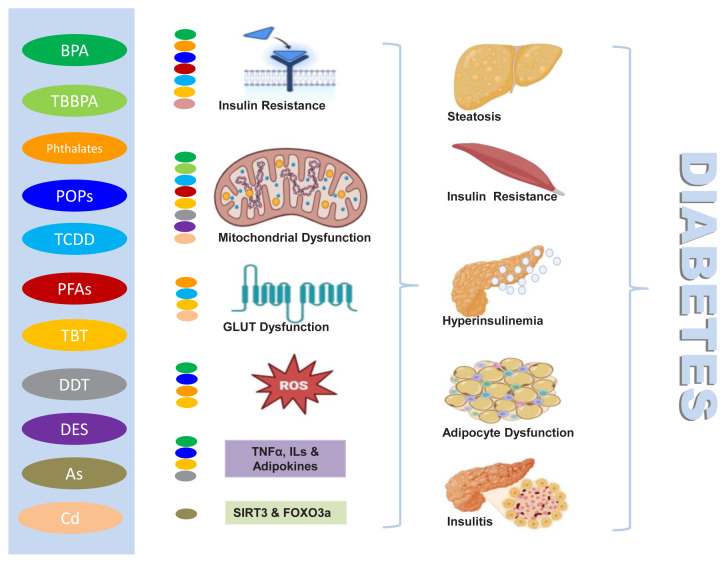
Schematic presentation of the main EDCs with the potential mechanisms for the occurrence of diabetes. This figure illustrates the potential mechanisms by which the main EDCs discussed in this article may contribute to the development of diabetes. Each EDC shown in the diagram is represented by a distinct colour. BPA: bisphenol A; TBBPA: tetrabromobisphenol A; POPs: persistent organic pollutants; TCDD: 2,3,7,8-tetrachlorodibenzo-p-dioxin; PFAS: perfluoroalkyl acids; TBT: tributyltin; DDT: dichlorodiphenyltrichloroethane; DES: diethylstilbestrol; As: arsenic; Cd: cadmium; GLUT: glucose transporter; ROS: reactive oxygen species; TNF: tumour necrosis factor; ILs: interleukins; SIRT3-FOXO3a: sirtuin 3-forkhead box O3 pathway.
